# Refined Cumulative Risk Assessment of Pb, Cd, and as in TCM Decoction Based on Bioavailability through In Vitro Digestion/MDCK Cells

**DOI:** 10.3390/toxics12070528

**Published:** 2024-07-22

**Authors:** Tiantian Zuo, Feiya Luo, Yaqiong Suo, Yan Chang, Zhao Wang, Hongyu Jin, Jiandong Yu, Shuxia Xing, Yuansheng Guo, Dandan Wang, Feng Wei, Gangli Wang, Lei Sun, Shuangcheng Ma

**Affiliations:** 1 National Key Laboratory of Drug Regulatory Science, National Institutes for Food and Drug Control, Beijing 100050, China; zuotiantian2011@163.com (T.Z.); luofeiya@nifdc.org.cn (F.L.); suoyq@nifdc.org.cn (Y.S.); changyan@nifdc.org.cn (Y.C.); wangzhao@nifdc.org.cn (Z.W.); jinhongyu@nifdc.org.cn (H.J.); yujiandong@nifdc.org.cn (J.Y.); xingshuxia@nifdc.org.cn (S.X.); gysheng2022@163.com (Y.G.); wangdandan@nifdc.org.cn (D.W.); wanggl@nifdc.org.cn (G.W.); sunlei@nifdc.org.cn (L.S.); 2 Chinese Pharmacopeia Commission, Beijing 100061, China

**Keywords:** bioavailability, traditional Chinese medicines, decoction, cumulative risk assessment, in vitro digestion/MDCK cell, heavy metal(loid)s

## Abstract

In this study, the oral bioavailability of Pb, Cd, and As in three types of traditional Chinese medicines (TCMs) and TCM decoctions were investigated through in vitro PBET digestion/MDKC cell model. Furthermore, a novel cumulative risk assessment model associated with co-exposure of heavy metal(loid)s in TCM and TCM decoction based on bioavailability was developed using hazard index (HI) for rapid screening and target organ toxicity dose modification of the HI (TTD) method for precise assessment. The results revealed that the bioavailability of Pb, Cd, and As in three types of TCM and TCM decoction was 5.32–72.49% and 4.98–51.97%, respectively. After rapid screening of the co-exposure health risks of heavy metal(loid)s by the HI method, cumulative risk assessment results acquired by TTD method based on total metal contents in TCMs indicated that potential health risks associated with the co-exposure of Pb, Cd, and As in *Pheretima aspergillum* (E. Perrier) and *Oldenlandia diffusa* (Willd.) Roxb were of concern. However, considering both the factors of decoction and bioavailability, TTD-adjusted HI outcomes for TCMs in this study were <1, indicating acceptable health risks. Collectively, our innovation on cumulative risk assessment of TCM and TCM decoction provides a novel strategy with the main purpose of improving population health.

## 1. Introduction

With a lengthy history of use, traditional Chinese medicine (TCM) has garnered attention for its positive pharmacological effects and remarkable therapeutic properties [1,2,3]. Nonetheless, TCM may suffer contamination by heavy metal(loid)s during cultivation, harvesting, and processing [4]. The ingestion of heavy metal-contaminated TCM through the food chain results in their accumulation in humans, posing detrimental effects on public health. Considerable attention has been placed on heavy metal(loid)s owing to their adverse impact on living organisms, including humans [5,6,7]. Lead (Pb), cadmium (Cd), and arsenic (As) are recognized as detrimental to human health, even at minimal concentrations [8,9,10,11]. Previous research has highlighted the harmful impact of heavy metal(loid)s, involving altering DNA functions, cell signaling, and protein folding [12,13]. Prolonged exposure to heavy metal(loid)s leads to reproductive deficiencies, disruptions in the regular neurological or reproductive function, and an elevated risk of cancer [14,15,16].

TCMs are usually consumed after decoction, with only decoction-transferred toxicants being absorbable by the human body. In practice, not all TCM heavy metal contents are fully assimilated. Hence, assessing bioaccessible and bioavailable heavy metal content is crucial, as they more directly correlate with toxicity than total contents in TCMs. Bioaccessible content refers to the level of pollutants capable of being released from the matrix into the intestinal tract, with the potential for absorption during human digestion [17,18,19,20,21]. Recently, diverse in vitro digestion methods have been implemented to assess the bioaccessibility of harmful elements in soil and food. These methods include in vitro PBET (Physiologically Based Extraction Test), SBET (Simplified bioaccessibility exaction test), RIVM (Rijksinstituut voorVolksgezondheid en Milieu), and SHIME (Simulator of human intestinal microbial ecosystem), among others [22,23,24,25,26]. In vitro digestion models offer significant benefits, including timeliness, simplicity, cost-effectiveness, energy efficiency, and ease of reproducibility [7,27]. Bioavailable content represents the level absorbed by the body, directly contributing to physiological processes. Therefore, it provides a more genuine indication of oral pathway exposure [27,28,29,30,31]. The Madin–Darby canine kidney (MDCK) cell line, originating from the collecting duct of the canine kidney, is a well-recognized secretory epithelial cell line commonly employed as an in vitro means for evaluating drug absorption and metabolism, which is endorsed by regulatory agencies such as the Food and Drug Administration (FDA), European Medicines Agency (EMA), and other authorities.

Common health risk assessment methods for heavy metal(loid)s often focus on individual pollutant chemicals, providing valuable insights into the preliminary evaluation of specific chemical risks. Nevertheless, real-life scenarios involve human exposure to diverse pollutants through numerous pathways. The simultaneous exposure to various pollutants through diverse pathways and media is termed cumulative exposure. Assessing the health risk associated with cumulative exposure is referred to as cumulative risk assessment, presenting scientists with challenges related to the toxicological consequences of mixtures and the development of suitable risk assessment methodologies [32,33].

Limited data exist on the bioavailable contents of heavy metal(loid)s in TCM decoctions, and scant information has been published on the cumulative health risks associated with these bioavailable heavy metal(loid)s. Therefore, establishing models to detect bioavailable heavy metal contents in TCM decoctions and formulating a cumulative health risk assessment methodology is of great significance to uniform the clinical usage of TCMs. Given the widespread adoption of TCMs globally and the respective public health apprehensions, the present work has focused on (1) analyzing heavy metal levels in TCMs via inductively coupled plasma mass spectroscopy (ICP-MS); (2) uncovering bioavailable heavy metal contents in both TCMs and their decoctions through human in vitro digestion simulations (for this purpose, in vitro digestion/MDCK cell models were employed); (3) constructing a cumulative risk assessment approach for TCMs and their decoctions based on exposure to bioavailable heavy metal(loid)s. This comprehensive strategy aims to offer succinct and original insights into health risks while promoting the safe use of TCMs.

## 2. Materials and Methods

### 2.1. Samples and Reagents

A total of three types of TCMs involving *Pheretima aspergillum* (E. Perrier), *Curcuma kwangsiensis* (S. G. Lee et C. F. Liang), and *Oldenlandia diffusa* (Willd.) Roxb were collected (Table 1). The samples were authenticated by Dr. Shuai Kang, an expert on the identification of medicinal materials at the National Institutes for Food and Drug Control (NIFDC). Double-deionized water was prepared using a Milli-Q water purification system (Millipore, Milford, MA, USA). The Pb, Cd, and As mono-standard solutions were purchased from the National Standard Material Research Center (Beijing, China). The internal standard solution and tuning solutions containing Li, Y, Ce, Tl, and Co were obtained from Agilent (Agilent Technologies, Folsom, CA, USA). Supra-pure trace metal-grade concentrated nitric acid (HNO_3_, 65.0%) was purchased from Merck (Merck, Munchen, Germany). Unless otherwise stated, chemicals for in vitro PBET digestion were purchased from Sigma Chemical (St. Louis, MO, USA). MDCK cells were purchased from Shanghai Institutes for Biological Science of the Chinese Academy of Medical Sciences (Shanghai, China). For cell cultures associated with MDCK models, fetal bovine serum (FBS), Dulbecco’s modified Eagle’s medium (DMEM) containing glucose, and 0.25% trypsin/EDTA were obtained from Gibco (Carlsbad, CA, USA).

### 2.2. Preparation of TCM Decoction

Homogenous TCM powder (5 g) was taken into a beaker and decocted with 40 mL of water for 30 min. The resulting suspension was filtered. The decoction process was repeated twice. The filtered liquor was combined and concentrated to 2 mL, and then transferred to a 5 mL volumetric flask, where it was brought to the scale by double-deionized water, constituting the final TCM decoction.

### 2.3. Determination of the Heavy Metal Concentrations in TCM and TCM Decoction Using ICP-MS

Processing 0.5 g of homogenous TCM powder or TCM decoction involved transferring it into microwave digestion vessels and subsequent digestion with HNO_3_ (5 mL). Following digestion, excess acid in the vessels was expelled. The resulting digestion solution was diluted with Milli-Q water until reaching 50 mL. Heavy metal concentrations were assessed via ICP-MS, applying our previously published procedures [21]. To ensure quality, blanks and triplicates were executed throughout the determination workflow. To counteract signal drift and matrix effects, an internal standard was introduced to blanks, samples, and calibration standards. The internal standard solution demonstrated a mean recovery rate ranging from 92.5% to 108.6%. Method accuracy was monitored through the mean recovery rate of the samples (*n* = 6). The mean percentage recoveries of heavy metal(loid)s in *Curcuma kwangsiensis* (S. G. Lee et C. F. Liang) were as follows: 92.4 ± 1.2% (Pb), 103.7 ± 0.7% (Cd), and 82.9 ± 4.3% (As). The mean percentage recoveries of heavy metal(loid)s should be in the range of 80% to 120%, which indicated that our method is accurate. The closer the recovery rate to 100%, the more accurate the method is. The recovery rate of As is exactly in the range of 80% to 120%, but less than 100%, which may lead to a slightly underestimated risk of As.

### 2.4. In Vitro Digestion

The investigation into the bioaccessible contents of heavy metal(loid)s in both TCM and TCM decoction employed the in vitro PBET model, following our documented protocols [21]. This model comprises two compartments, encompassing gastric and intestinal extraction phases. Briefly, during the gastric extraction phase, TCM or TCM decoction was blended with a simulated gastric solution of pH 2.0 (prepared with HCl) to a volume of 50 mL. Subsequently, the mixed solutions were taken to incubation at 37 °C, agitated for 1 h, and centrifuged for 5 min to obtain the supernatant. Following this, nitric acid (5 mL) was added to the concentrated supernatant (3 mL) to trigger digestion. Ultimately, the 50 mL digested solution, representing the gastric portion, was subjected to analysis by ICP-MS.

During the phase of intestinal extraction, the remains obtained from the gastric extraction phase were introduced into a simulated intestinal solution comprising pancreatin and bile salts (with a pH of 7.0) within a 50 mL capacity. This mixture underwent incubation and shaking at 37 °C for 4 h, followed by centrifugation to gather the supernatant, which was subsequently condensed to a volume of 3 mL. Subsequently, 5 mL of HNO_3_ was incorporated into the concentrated supernatant to facilitate digestion. In the final step, the 50 mL digested solution representing the intestinal fraction was determined using ICP-MS.

### 2.5. MDCK Cell Model

MDCK cells were kept in DMEM with a 10% (*v*/*v*) FBS supplementation and 2.3 g/L sodium bicarbonate. Cell incubation was conducted under 95% air and 5% CO_2_ at 37 °C. Every 2 days, the culture medium was renewed. Cell transfer was executed when the cell density totaled 80% confluence via 0.25% trypsin/EDTA addition. For cell differentiation and uptake assessments, a two-chamber well (basolateral and apical) with a polyester membrane of 12 mm diameter and 0.4 μm pore size (Millipore, Burlington, MA, USA) was utilized. MDCK cells were seeded at a density of 4.0 × 10^5^ cells/mL, respectively. In order to foster the maturation of cells and the development of differentiated confluent cell monolayers, MDCK cells were subjected to a 4-day incubation period, with the culture medium undergoing refreshment every 2 days. The assessment of cell monolayer integrity relied on measuring transepithelial electrical resistance (TEER) using Millicell ERS (WPI Corporation, USA). Only monolayers with TEER values surpassing 200 Ω/cm^2^ were considered for the subsequent uptake tests. The uptake examinations for MDCK cells were executed 4 days after seeding. A 0.5 mL aliquot of the intestinal fraction was introduced to the apical side, while 0.5 mL of FBS-free DMEM was administered to the basolateral side. Following a 4 h incubation (37 °C, 5% CO_2_), the solutions from the apical and basal compartments were separately collected to quantify the transport of Pb, Cd, and As throughout the cell monolayer. Subsequently, cell monolayers underwent lysis and microwave digestion. The heavy metal content within the cell lysate was prepared for analysis by employing ICP-MS for retention test. Control cells were included in each assay to establish a baseline.

The quantification of heavy metal cellular uptake was represented by the accumulation of heavy metal(loid)s absorbed by MDCK cells (comprising both retention and transport) from the intestinal fraction, divided by the heavy metal content present in the intestinal fraction. The calculation of heavy metal uptake was determined through the application of the following formula:

(1) $$\mathrm{Uptake}\;(\%)\;=\;\frac{heavy\;metals\;in\;basolateral\;+\;heavy\;metals\;in\;cell\;monolayer}{heavy\;metals\;in\;intestinal\;fraction}\times 100\%$$

## 3. Health Risk Assessment

### 3.1. Risk Assessment Based on the HQ Method

To analyze the health risks due to the ingestion of one toxic metal in TCM and TCM decoction, hazard quotient (HQ) was calculated using Equation (2) [34]:

(2) $$HQ=\frac{EF\times Ed\times IR\times C\times S\times BA}{W\times AT\times RfD}$$

Based on our data, the exposure duration (Ed), the exposure frequency (EF), and the safety factor (S) for TCM were recorded as 20 years, 90 days per year, and 10, respectively. Additionally, the average time exposed to TCMs (AT) and average body weight (W) were noted as 365 days per year multiplied by 70 years and 60 kg, respectively [34]. IR denotes the ingestion rate of TCMs, aligning with the maximum daily ingestion rate stipulated in the Chinese Pharmacopoeia. C represents the total contents in TCM or TCM decoction (mg/kg). BA was the bioavailability of heavy metal(loid)s, and was calculated using Equation (3). The oral reference dose values (RfD) recommended for Pb, Cd, and As were established at 3.5, 1, and 0.3 μg/kg bw/day, respectively [35].

$$\mathrm{Bioavailability}\;(\%)\;=\;\frac{bioaccessible\;heavy\;metal\;contents\;in\;gastric\;phase\;+\;bioaccessible\;heavy\;metal\;contents\;after\;uptake}{total\;heavy\;metal\;contents}\times 100\%$$

### 3.2. Preliminary Cumulative Risk Assessment Using Hazard Index

The potential additive effects arising from exposure to two or more toxic chemicals were evaluated through a preliminary cumulative risk evaluation via the hazard index (HI) approach. This assessment followed the classical approach, using the equation below:

HI = HQ_Pb_ + HQ_Cd_ + HQ_As_ (3)

### 3.3. Accurate Cumulative Risk Evaluation Utilizing Target Organ Toxicity Dose (TTD) Modification of HI Method

The TTD adjustment of the HI method was devised to accommodate the assessment of mixtures with components manifesting different critical effects but sharing overlapping organs of toxicity. Under this modification, chemicals with multiple target organs have corresponding toxicity doses, expressed as precise health-based guidance values (PHBGVs) for the evaluation of joint toxic actions of Pb, Cd, and As in TCM decoction. Specific-end-point hazard quotients (HQs) were computed using Equation (2) to derive the TTD-modified HI values. In accordance with the guidelines set forth by ATSDR, the PHBGVs of Pb for hematological, cardiovascular, neurological, testicular, and renal systems were 4.2, 1.3, 4.2, 16.7, and 0.6 μg/kg bw/day, respectively. The PHBGVs of Cd for hematological, cardiovascular, neurological, testicular systems, and renal were 0.80, 5.00, 0.20, 3.00, and 0.83 μg/kg bw/day, respectively. Additionally, the PHBGVs for As concerning hematological, cardiovascular, renal, and neurological systems were 0.60, 0.30, 90.00, and 0.30 μg/kg bw/day, respectively [36].

If HI or TTD modification of HI is below 1, it suggests that there are no anticipated significant adverse health effects from exposure to TCM or TCM decoction. Conversely, if HI or TTD modification of HI exceeds 1, the health risk for the exposed population should not be disregarded. 

## 4. Results

### 4.1. Concentration of Heavy Metal(loid)s in TCMs

**Figure 1:**
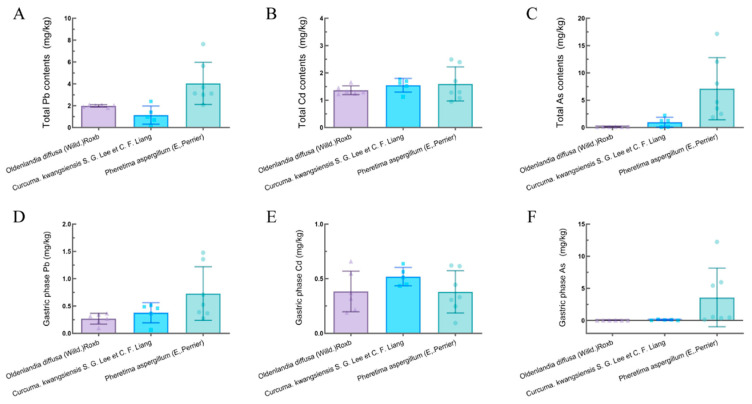
Contents of heavy metal(loid)s in 3 types of TCM. (**A**) Total contents of Pb. (**B**) Total contents of Cd. (**C**) Total contents of As. (**D**) Contents of Pb in gastric phase. (**E**) Contents of Cd in gastric phase. (**F**) Contents of As in gastric phase.

In general, the levels of Pb, Cd, and As in three types of TCMs showed wide variability, ranging from 0.210 to 7.641 mg/kg for Pb, 0.944 to 2.494 mg/kg for Cd, and 0.056 to 17.157 mg/kg for As (Figure 1A–C). Mean values indicated that among the three types of TCMs, *Pheretima aspergillum* (E. Perrier) demonstrated the most elevated accumulation of Pb, Cd, and As.

### 4.2. Concentration of Heavy Metal(loid)s in TCM Decoction

**Figure 2:**
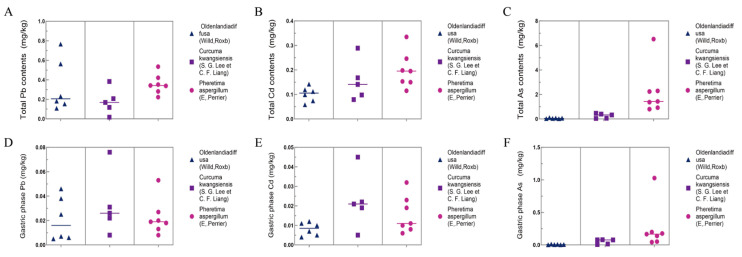
Contents of heavy metal(loid)s in 3 types of TCM decoction. (**A**) Total contents of Pb. (**B**) Total contents of Cd. (**C**) Total contents of As. (**D**) Contents of Pb in gastric phase. (**E**) Contents of Cd in gastric phase. (**F**) Contents of As in gastric phase.

The total amounts of Pb, Cd, and As in the TCM decoction ranged from 0.017 to 0.563, 0.058 to 0.335, 0.025 to 6.520, and 0.232 to 7.467 mg/kg, respectively (Figure 2A–C). Among the three types of TCMs, *Oldenlandia diffusa* (Willd.) Roxb had the highest total Pb content in the decoction, while *Curcuma kwangsiensis* (S. G. Lee et C. F. Liang) exhibited the lowest. *Pheretima aspergillum* (E. Perrier) had the highest total Cd and As contents in the decoction, with *Oldenlandia diffusa* (Willd.) Roxb showing the lowest. Additionally, *Pheretima aspergillum* (E. Perrier) had the highest total Cu content in the decoction, while *Curcuma kwangsiensis* (S. G. Lee et C. F. Liang) had the lowest.

### 4.3. Bioaccessible Amounts and Bioavailability of Heavy Metal(loid)s in TCM

The bioaccessible amounts of Pb, Cd, and As in the gastric phase (G) ranged from 0.064 to 1.477, 0.094 to 0.659, and 0.008 to 2.230 mg/kg, respectively (Figure 1D–F). Among the three types of TCMs, *Curcuma kwangsiensis* (S. G. Lee et C. F. Liang) exhibited the highest bioaccessible contents of Cd in the gastric phase, while *Pheretima aspergillum* (E. Perrier) showed the highest bioaccessible contents of Pb and As. Conversely, *Oldenlandia diffusa* (Willd.) Roxb had the lowest bioaccessible contents of Pb and As, while *Pheretima aspergillum* (E. Perrier) exhibited the lowest bioaccessible levels of Cd.

To enhance the accuracy of estimating heavy metal uptake in the intestine, the in vitro PBET digestion was coupled with MDCK cell cultures, considering that absorption in intestinal epithelial cells can diminish the portion of heavy metal(loid)s reaching the bloodstream. 

Another model, the Caco-2 cell model, may currently be considered the most commonly used in vitro model for evaluating cell bioavailability. However, the Caco-2 cell model lacks the protection afforded by the intestinal mucus layer that is present in the human intestinal environment, and accordingly, may undergo degradation by enzymes in digestive fluids, leading to compromised evaluation data. MDCK cells, which are typical secretory epithelial cell lines originally from the collecting duct of the canine kidney, are also widely recognized as an in vitro tool for evaluating drug absorption and metabolism [37,38,39,40]. In addition, MDCK cells have the following advantages: short cultivation time, stability, and accuracy. Therefore, the MDCK cell line was applied in this study.

**Figure 3:**
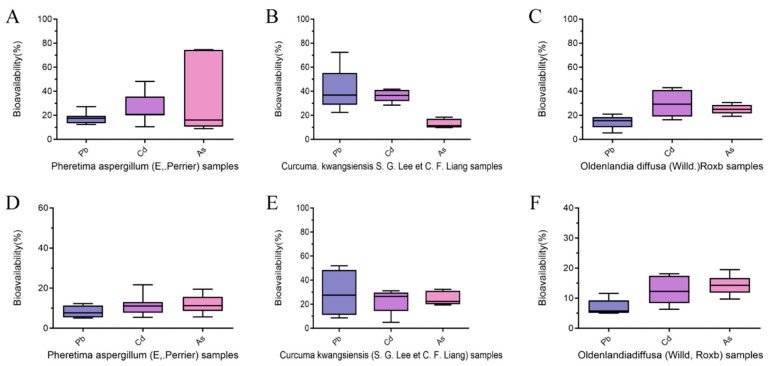
Bioavailability of heavy metal(loid)s in 3 types of TCM and TCM decoction. (**A**) Bioavailability of heavy metal(loid)s in *Pheretima aspergillum* (E. Perrier). (**B**) Bioavailability of heavy metal(loid)s in *Curcuma kwangsiensis* (S. G. Lee et C. F. Liang). (**C**) Bioavailability of heavy metal(loid)s in *Oldenlandia diffusa* (Willd.) Roxb. (**D**) Bioavailability of heavy metal(loid)s in decoction of *Pheretima aspergillum* (E. Perrier). (**E**) Bioavailability of heavy metal(loid)s in decoction of *Curcuma kwangsiensis* (S. G. Lee et C. F. Liang). (**F**) Bioavailability of heavy metal(loid)s in decoction of *Oldenlandia diffusa* (Willd.) Roxb.

The bioavailability after the uptake of Pb in MDCK cells in *Pheretima aspergillum* (E. Perrier), *Curcuma kwangsiensis* S. G. Lee et C. F. Liang, and *Oldenlandia diffusa* (Willd.) Roxb. was in the range of 12.39–27.21%, 22.38–72.49%, and 5.32%-21.02%, respectively (Figure 3A–C). The bioavailability after the uptake of Cd in MDCK cells in *Pheretima aspergillum* (E. Perrier), *Curcuma kwangsiensis* S. G. Lee et C. F. Liang, and *Oldenlandia diffusa* (Willd.) Roxb. was in the range of 10.57–48.30%, 28.45–41.75%, and 16.27–43.07%, respectively. The bioavailability after the uptake of As in MDCK cells in *Pheretima aspergillum* (E. Perrier), *Curcuma kwangsiensis* S. G. Lee et C. F. Liang, and *Oldenlandia diffusa* (Willd.) Roxb. was in the range of 8.97–16.09%, 9.79–18.55%, and 19.21–30.74%, respectively.

#### Bioaccessible Contents and Bioavailability of Heavy Metal(loid)s in TCM Decoction

The bioaccessible amounts of Pb, Cd, and As pertaining to the gastric phase (G) were 0.005–0.076, 0.005–0.045, and 0.002–1.028 mg/kg, respectively (Figure 2D–F). The bioavailability after the uptake of Pb in MDCK cells in *Pheretima aspergillum* (E. Perrier), *Curcuma kwangsiensis* (S. G. Lee et C. F. Liang), and *Oldenlandia diffusa* (Willd.) Roxb was in the range of 5.09–12.30%, 8.65–51.97%, and 4.98–11.57%, respectively (Figure 3D–F). The bioavailability after the uptake of Cd in MDCK cells in *Pheretima aspergillum* (E. Perrier), *Curcuma kwangsiensis* (S. G. Lee et C. F. Liang), and *Oldenlandia diffusa* (Willd.) Roxb was in the range of 5.42–21.67%, 4.95–31.13%, and 6.30–18.10%, respectively. The bioavailability after the uptake of As in MDCK cells in *Pheretima aspergillum* (E. Perrier), *Curcuma kwangsiensis* (S. G. Lee et C. F. Liang), and *Oldenlandia diffusa* (Willd.) Roxb was in the range of 5.66–19.46%, 19.39–32.26%, and 9.73–19.49%, respectively.

## 5. Health Risk Assessment

### 5.1. Health Risk Assessment for a Single Metal

**Figure 4:**
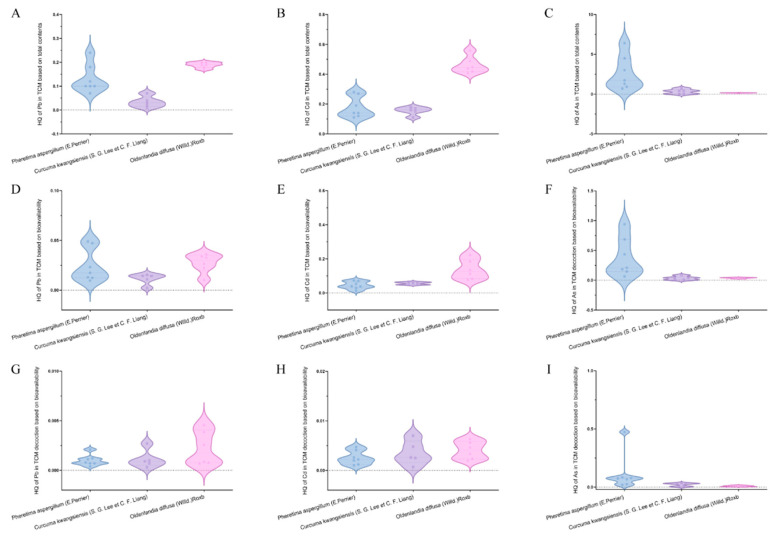
HQ for 3 types of TCM and TCM decoction. (**A**) HQ of Pb for TCM based on total contents. (**B**) HQ of Cd for TCM based on total contents. (**C**) HQ of As for TCM based on total contents. (**D**) HQ of Pb for TCM based on bioavailability. (**E**) HQ of Cd for TCM based on bioavailability. (**F**) HQ of As for TCM based on bioavailability. (**G**) HQ of Pb for TCM decoction based on bioavailability. (**H**) HQ of Cd for TCM decoction based on bioavailability. (**I**) HQ of As for TCM decoction based on bioavailability.

To assess possible health risks, HQs for heavy metal(loid)s in TCM were computed using both total amounts and bioavailable amounts after transportation by MDCK cells, denoted as HQt and HQb, respectively (Figure 4A–F). Overall, the findings indicated that HQt values for TCMs were significantly higher compared to those calculated using the bioavailable levels of TCM decoction.

In the case of using TCM as raw powder, levels of As were evaluated and the results indicated that HQ_t_ values for five batches of *Pheretima aspergillum* (E. Perrier) exceeded 1, signaling an unacceptable health risk. Nevertheless, when bioavailability was considered based on in vitro digestion/MDCK cell models, the HQ_b_ values for Pb, Cd, and As were below 1 for all TCMs, signifying a tolerable health risk. For TCM used after decoction, HQs of heavy metal(loid)s in TCM decoction underwent calculation based on the bioavailable amounts after transportation by MDCK cells, denoted as HQ_d_ (Figure 4G–I). As a result, when considering both the factor of decoction and bioavailability, Pb, Cd, and As had HQ_d_ values below 1 for each type of TCM, indicating a negligible health risk.

### 5.2. Preliminary Cumulative Risk Assessment Based on Hazard Index

In the scenario where TCMs were used as raw powder, the assessment of total Pb, Cd, and As amounts indicated HI values exceeding 1 for six batches of *Pheretima aspergillum* (E. Perrier), signaling the need for attention due to potential cumulative health risks from co-exposure of Pb, Cd, and As. However, when utilizing bioavailable amounts of Pb, Cd, and As, it was observed that the HI outcomes for all these TCMs were below 1. This result suggested that the associated health effects of Pb, Cd, and As were acceptable. In the scenario where TCMs were used after decoction, the HI outcomes for all three sorts of TCMs were consistently below 1. This indicated that co-exposure of Pb, Cd, and As in TCM decoction may not lead to harmful systemic effects.

### 5.3. Precise Cumulative Risk Assessment According to TTD

#### In the Scenario Where TCMs Were Used as Raw Powder

In the scenario where TCMs were used as raw powder, the distinct toxic effects of Pb, Cd, and As on various organs necessitate a precise cumulative risk assessment. The cardiovascular system emerges as the most sensitive target organ for Pb, while the kidney, with β2-microglobulin as the marker, is the most sensitive end-point for Cd, and the lung is the most vulnerable organ for As. This study explored a more precise cumulative risk assessment considering these diverse toxic target organs. When TCMs were used as raw power, the evaluation of total Pb, Cd, and As contents in TCMs revealed that TTD-modified HI values for all batches of *Pheretima aspergillum* (E. Perrier) exceeded 1 for the cardiovascular system (Table 2). In the hematological aspect, four batches of *Pheretima aspergillum* (E. Perrier) exhibited TTD-modified HIs surpassing 1. For the neurological system, TTD-modified HI values for three batches of *Curcuma kwangsiensis* (S. G. Lee et C. F. Liang), all batches of *Oldenlandia diffusa* (Willd.) Roxb, and *Pheretima aspergillum* (E. Perrier) went beyond 1. Concerning the renal outcome, TTD-modified HI values for all batches of *Oldenlandia diffusa* (Willd.) Roxb and two batches of *Pheretima aspergillum* (E. Perrier) exceeded 1. These TTD-modified HI outcomes suggested that, excluding two batches of *Curcuma kwangsiensis* (S. G. Lee et C. F. Liang), the health hazards tied to the combination of Pb, Cd, and As in these three TCM types might surpass the human tolerance threshold. Nevertheless, when incorporating bioavailable contents, the TTD-modified HI outcomes for one batch of *Oldenlandia diffusa* (Willd.) Roxb and two batches of *Pheretima aspergillum* (E. Perrier) exceeded 1 for the neurological system. Notably, the TTD-modified HI values based on bioavailable contents were markedly inferior to those computed using the total contents in TCMs.

### 5.4. In the Scenario Where TCMs Were Used after Decoction

In the context of TCM use after decoction, utilizing bioavailable levels of Pb, Cd, and As, the TTD-modified HI outcomes for all types of TCM showed to be consistently below 1 for the hematological, cardiovascular, neurological, testicular, and renal systems, signifying a tolerable health risk (Table 3). Bioavailability assumes a pivotal role in health risk evaluations, offering a precise assessment model for toxins. Furthermore, it aids in averting the overestimation of health hazards, and it thus prevents unnecessary government involvement and the overconsumption of TCM resources. As far as we know, this study pioneers a meticulous cumulative risk assessment of heavy metal(loid)s based on bioavailability considering diverse TCM usage scenarios, especially the scenario where TCMs were used after decoction. This innovative and precise health risk estimation strategy, incorporating bioavailability characterization, supports the safety of ingesting *Curcuma kwangsiensis* (S. G. Lee et C. F. Liang), *Oldenlandia diffusa* (Willd.) Roxb, and *Pheretima aspergillum* (E. Perrier) after decoction.

## 6. Discussion

Not all substances are released from matrices into the systemic circulation during digestion. Therefore, the bioavailability usually remains below 100%, and assessments of the health risk of heavy metal(loid)s based on their total amounts in TCM will lead to an overestimation of the harm posed to the human body. This may result in the adoption of excessively stringent regulatory measures by the government. Few studies have investigated the bioaccessibility of heavy metal(loid)s in the TCM decoction. In the field of food research, it has been found that the bioaccessibility of heavy metal(loid)s in vegetables is influenced by the cooking methods, physicochemical properties of the elements, and even gut microorganisms. For example, studies performed using the in vitro PBET model demonstrated that the selection of appropriate cooking methods could significantly reduce the bioaccessibility of heavy metal(loid)s in vegetables [41,42]. Differences also exist in bioaccessibility determined using different models. For instance, the bioaccessibility of Cd in rice obtained using the in vitro PBET model was 30% to 50%, slightly lower than the values obtained with the RIVM model (74% to 83% and 90.04% to 100.70%) [42,43]. Torres-Escribano utilized two in vitro gastrointestinal digestion methods: a static method (SM) and a dynamic multicompartment method (TIM-1) to measure the bioaccessibility of As, Cd, Pb, and Hg in seaweed. Significant differences were observed between the bioaccessibility values obtained from the SM and TIM-1 models. [44]. This is likely attributed to the combined influence of various in vitro conditions (e.g., pH, digestion time, enzyme concentration and type, solid–liquid ratio, dynamic and static modulation of peristalsis).

The in vitro PBET method, first proposed by Dr. Ruby et al. in 1996, involves an in vitro experiment performed through simulation of two digestive phases of the human body, namely the gastric and small intestinal phases. In the present study, we selected the in vitro PBET method, as it entails the addition of pepsin and organic acids to the digestive fluid during the simulation of the gastric digestive environment and the addition of bile and trypsin when simulating the small intestinal digestive environment. This results in a more realistic simulation of the physiological environment of the human body. And considering that bioaccessibility is associated with the maximum oral bioavailability and absorption in the intestinal cells decreases the portion of heavy metal(loid)s that reaches the body circulation, the in vitro PBET digestion was advanced by coupling with MDCK cell cultures to offer a more accurate estimate of the uptake in the intestine in the present study. The bioavailability and uptake of heavy metal(loid)s by MDCK cells were influenced by many factors. Firstly, the uptake efficiency is directly related to the gene expression of heavy metal(loid)s in cells. For example, the increased uptake of heavy metal(loid) was attributed to the upregulation of MRP1 gene expression [45]. Additionally, the uptake of heavy metal(loid)s is associated with the activity of the ionic form in solution [46]. Moreover, the pH, temperature, and other environmental factors greatly influence the uptake of heavy metal(loid)s [47]. In addition, the uptake of heavy metal(loid)s can be evidently modulated by the organism’s requirement for essential metals, including Zn, Fe, and Ca. When these nutrient elements are in short supply, the accumulation and toxicity of heavy metal(loid)s are enhanced [47].

Cumulative exposure assessments aim to ensure a comprehensive evaluation of a specific population’s exposure to various compounds through different routes within a certain time period. It is crucial to analyze the toxicity end-points of chemicals and consider the duration and frequency of exposure to different compounds. The two main types of exposure assessment models were designed for preliminary risk screening and precise assessment, each requiring distinct toxicological parameters with different scopes of application. The hazard index (HI) method, though simple and rapid, is more suitable for initial risk screening. To realize precise risk assessments, the United States Environmental Protection Agency (US EPA) recommends further grouping compounds by the mechanism of action or target consistency, considering all possible effects or target organs that may be affected. In this study, we initially employed the HI method for the initial screening of combined exposure risk to heavy metal(loid)s in three TCMs and TCM decoctions. Subsequently, we delved deeper using the higher-level target organ toxicity dose (TTD) approach to precisely assess potential health risks associated with Pb, Cd, and As. By incorporating bioavailability into the assessment model, we hope that our findings provide innovative approaches for developing risk assessment methods for heavy metal(loid)s in TCM and TCM decoction. In further study, the stochastic behavior of the risk model would be explored using the probability distribution of inputs, random numbers, and statistical sampling methods to explore the health risks of different exposure populations [48].

## 7. Conclusions

In this study, we introduced an innovative approach to assess the cumulative health risks due to co-exposure of heavy metal(loid)s in TCMs and TCM decoctions, utilizing in vitro PBET digestion/MDKC cell models. This approach considered both the decoction factor and bioavailability, marking the first instance of such a comprehensive evaluation. Our aim was to establish scientifically precise assessment methods for determining the health risks associated with heavy metal(loid)s in TCM and TCM decoctions. Our primary findings underscore the importance of evaluating health risks based on bioavailable metal amounts rather than total amounts in TCMs, particularly in diverse exposure scenarios involving both raw powder and decoction. This approach ensures a more realistic and precise measurement of potential toxins to humans, mitigating the risk of overestimation and preventing unnecessary waste of medicinal resources. It is crucial to note that the calculations should not overlook the potential for cumulative health risks. We anticipate that our novel cumulative health risk assessment approach, valid for both TCM and TCM decoctions, utilizing in vitro digestion/cellular uptake, will contribute valuable insights into the bioaccumulation of heavy metal(loid)s in organisms and advance the toxicology assessment of TCM and TCM decoctions, with the main purpose of improving public health.

## Figures and Tables

**Table 1:** Sample collection information in this study.

Type	No.	Batch No.	Location	Source
*Pheretima aspergillum* (E. Perrier)	1	DL-01	Anhui	TCM market
	2	DL-02	Anhui	TCM market
	3	DL-03	Shanghai	Pharmacy
	4	DL-04	Shanghai	Pharmacy
	5	DL-05	Shanghai	TCM market
	6	DL-06	Guangxi	TCM market
	7	DL-07	Guangdong	TCM market
*Curcuma kwangsiensis* S. G. Lee et C. F. Liang	1	EZ0-01	Guangxi	TCM market
	2	EZ-02	Guangxi	TCM market
	3	EZ-03	Guangxi	Pharmacy
	4	EZ-04	Yunnan	TCM market
	5	EZ-05	Fujian	Pharmacy
*Oldenlandia diffusa* (Willd.) Roxb	1	BHSSC-01	Zhejiang	TCM market
	2	BHSSC-02	Zhejiang	TCM market
	3	BHSSC-03	Yunnan	Pharmacy
	4	BHSSC-04	Fujian	Pharmacy
	5	BHSSC-05	Guangxi	Pharmacy
	6	BHSSC-06	Guangxi	TCM market

**Table 2:** Cumulative exposure assessment results of Pb, Cd, and As in TCMs based on TTD method.

Pb		Batch No.	Total					Bioavailability				
			Cardiovascular	Blood	Nerve	Kidney	Testis	Cardiovascular	Blood	Nerve	Kidney	Testis
	*Pheretima aspergillum* (E. Perrier)	1	0.26	0.08	0.08	0.56	0.02	0.03	0.01	0.01	0.08	2.70 × 10^−3^
		2	0.27	0.08	0.08	0.58	0.02	0.05	0.01	0.01	0.10	3.65 × 10^−3^
		3	0.66	0.20	0.20	1.43	0.05	0.13	0.04	0.04	0.28	9.89 × 10^−3^
		4	0.49	0.15	0.15	1.05	0.04	0.13	0.04	0.04	0.29	1.03 × 10^−2^
		5	0.18	0.05	0.05	0.38	0.01	0.03	0.01	0.01	0.06	2.01 × 10^−3^
		6	0.27	0.08	0.08	0.58	0.02	0.03	0.01	0.01	0.07	2.60 × 10^−3^
		7	0.32	0.10	0.10	0.69	0.02	0.06	0.02	0.02	0.14	4.84 × 10^−3^
	*Curcuma kwangsiensis* S. G. Lee et C. F. Liang	1	0.11	0.03	0.03	0.24	0.01	0.04	0.01	0.01	0.08	2.98 × 10^−3^
		2	0.19	0.06	0.06	0.40	0.01	0.04	0.01	0.01	0.09	3.24 × 10^−3^
		3	0.02	0.01	0.01	0.04	0.00	0.01	0.00	0.00	0.01	4.65 × 10^−4^
		4	0.08	0.02	0.02	0.17	0.01	0.03	0.01	0.01	0.06	2.26 × 10^−3^
		5	0.05	0.02	0.02	0.12	0.00	0.04	0.01	0.01	0.08	3.01 × 10^−3^
	*Oldenlandia diffusa* (Willd.) Roxb	1	0.52	0.16	0.16	1.13	0.04	0.06	0.02	0.02	0.13	4.75 × 10^−3^
		2	0.52	0.16	0.16	1.13	0.04	0.09	0.03	0.03	0.20	7.18 × 10^−3^
		3	0.54	0.17	0.17	1.17	0.04	0.09	0.03	0.03	0.19	6.92 × 10^−3^
		4	0.53	0.17	0.17	1.16	0.04	0.03	0.01	0.01	0.06	2.21 × 10^−3^
		5	0.47	0.14	0.14	1.01	0.04	0.10	0.03	0.03	0.21	7.65 × 10^−3^
		6	0.49	0.15	0.15	1.07	0.04	0.07	0.02	0.02	0.16	5.63 × 10^−3^
Cd	*Pheretima aspergillum* (E. Perrier)	1	0.02	0.15	0.61	0.15	0.04	2.55 × 10^−3^	0.02	0.06	0.02	0.00
		2	0.03	0.18	0.72	0.17	0.05	0.01	0.04	0.14	0.03	0.01
		3	0.06	0.35	1.40	0.34	0.09	0.01	0.09	0.37	0.09	0.02
		4	0.05	0.34	1.34	0.32	0.09	0.01	0.07	0.27	0.06	0.02
		5	0.04	0.24	0.95	0.23	0.06	0.01	0.05	0.20	0.05	0.01
		6	0.03	0.18	0.72	0.17	0.05	0.01	0.09	0.35	0.08	0.02
		7	0.02	0.13	0.53	0.13	0.04	0.01	0.05	0.19	0.05	0.01
	*Curcuma kwangsiensis* S. G. Lee et C. F. Liang	1	0.04	0.22	0.89	0.21	0.06	0.01	0.08	0.32	0.08	0.02
		2	0.03	0.21	0.82	0.20	0.05	0.01	0.07	0.29	0.07	0.02
		3	0.02	0.14	0.57	0.14	0.04	0.01	0.06	0.24	0.06	0.02
		4	0.03	0.19	0.77	0.19	0.05	0.01	0.08	0.31	0.08	0.02
		5	0.03	0.21	0.86	0.21	0.06	0.01	0.06	0.24	0.06	0.02
	*Oldenlandia diffusa* (Willd.) Roxb	1	0.09	0.56	2.23	0.54	0.15	0.03	0.17	0.68	0.16	0.05
		2	0.08	0.51	2.06	0.50	0.14	0.02	0.14	0.57	0.14	0.04
		3	0.10	0.61	2.43	0.59	0.16	0.02	0.10	0.40	0.1	0.03
		4	0.08	0.52	2.10	0.51	0.14	0.02	0.11	0.42	0.1	0.03
		5	0.11	0.70	2.78	0.67	0.19	0.04	0.28	1.12	0.27	0.07
		6	0.09	0.54	2.18	0.52	0.15	0.04	0.23	0.94	0.23	0.06
As	*Pheretima aspergillum* (E. Perrier)	1	1.30	0.65	1.30	4.34 × 10^−3^	/	0.21	0.11	0.21	6.98 × 10^−4^	/
		2	0.93	0.46	0.93	3.10 × 10^−3^	/	0.15	0.07	0.15	4.88 × 10^−4^	/
		3	4.50	2.25	4.50	1.50 × 10^−2^	/	0.69	0.34	0.69	2.28 × 10^−3^	/
		4	3.01	1.50	3.01	1.00 × 10^−2^	/	0.44	0.22	0.43	1.45 × 10^−3^	/
		5	6.41	3.20	6.41	2.14 × 10^−2^	/	0.94	0.47	0.94	3.13 × 10^−3^	/
		6	1.74	0.87	1.74	5.80 × 10^−3^	/	0.19	0.09	0.18	6.16 × 10^−4^	/
		7	0.70	0.35	0.70	2.34 × 10^−3^	/	0.06	0.03	0.06	2.10 × 10^−4^	/
	*Curcuma kwangsiensis* S. G. Lee et C. F. Liang	1	0.13	0.07	0.13	4.37 × 10^−4^	/	0.08	0.04	0.08	2.82 × 10^−4^	/
		2	0.16	0.08	0.16	5.49 × 10^−4^	/	0.01	3.53 × 10^−3^	0.01	2.35 × 10^−5^	/
		3	0.17	0.09	0.17	5.67 × 10^−4^	/	3.02 × 10^−3^	1.51 × 10^−3^	0.01	1.01 × 10^−5^	/
		4	0.13	0.06	0.13	4.18 × 10^−4^	/	0.04	0.02	0.04	1.38 × 10^−4^	/
		5	0.17	0.08	0.17	5.56 × 10^−4^	/	0.04	0.02	0.04	1.39 × 10^−4^	/
	*Oldenlandia diffusa* (Willd.) Roxb	1	0.74	0.37	0.74	2.48 × 10^−3^	/	0.03	0.02	0.03	1.08 × 10^−4^	/
		2	0.04	0.02	0.04	1.24 × 10^−4^	/	0.04	0.02	0.04	1.38 × 10^−4^	/
		3	0.02	0.01	0.02	6.27 × 10^−5^	/	0.04	0.02	0.04	1.27 × 10^−4^	/
		4	0.42	0.21	0.42	1.41 × 10^−3^	/	0.02	0.01	0.02	8.21 × 10^−5^	/
		5	0.41	0.21	0.41	1.38 × 10^−3^	/	0.05	0.02	0.05	1.57 × 10^−4^	/
		6	0.74	0.37	0.74	2.48 × 10^−3^	/	0.04	0.02	0.04	1.49 × 10^−4^	/

**Table 3:** Cumulative exposure assessment results of Pb, Cd, and As in TCM decoction based on bioavailability and TTD method.

Pb		Batch No.	Cardiovascular	Blood	Nerve	Kidney	Testis
	*Pheretima aspergillum* (E. Perrier)	1	1.98 × 10^−3^	6.13 × 10^−4^	6.13 × 10^−4^	4.29 × 10^−3^	1.54 × 10^−4^
		2	2.24 × 10^−3^	6.93 × 10^−4^	6.93 × 10^−4^	4.85 × 10^−3^	1.74 × 10^−4^
		3	5.69 × 10^−3^	1.76 × 10^−3^	1.76 × 10^−3^	1.23 × 10^−2^	4.43 × 10^−4^
		4	3.27 × 10^−3^	1.01 × 10^−3^	1.01 × 10^−3^	7.09 × 10^−3^	2.55 × 10^−4^
		5	2.84 × 10^−3^	8.80 × 10^−4^	8.80 × 10^−4^	6.16 × 10^−3^	2.21 × 10^−4^
		6	1.81 × 10^−3^	5.60 × 10^−4^	5.60 × 10^−4^	3.92 × 10^−3^	1.41 × 10^−4^
		7	9.48 × 10^−4^	2.93 × 10^−4^	2.93 × 10^−4^	2.05 × 10^−3^	7.38 × 10^−5^
	*Curcuma kwangsiensis* S. G. Lee et C. F. Liang	1	1.78 × 10^−3^	5.52 × 10^−4^	5.52 × 10^−4^	3.86 × 10^−3^	1.39 × 10^−4^
		2	2.56 × 10^−3^	7.92 × 10^−4^	7.92 × 10^−4^	5.54 × 10^−3^	1.99 × 10^−4^
		3	6.98 × 10^−4^	2.16 × 10^−4^	2.16 × 10^−4^	1.51 × 10^−3^	5.43 × 10^−5^
		4	7.21 × 10^−3^	2.23 × 10^−3^	2.23 × 10^−3^	1.56 × 10^−2^	5.61 × 10^−4^
		5	2.48 × 10^−3^	7.68 × 10^−4^	7.68 × 10^−4^	5.38 × 10^−3^	1.93 × 10^−4^
	*Oldenlandia diffusa* (Willd.) Roxb	1	1.24 × 10^−2^	3.84 × 10^−3^	3.84 × 10^−3^	2.69 × 10^−2^	9.66 × 10^−4^
		2	2.33 × 10^−3^	7.20 × 10^−4^	7.20 × 10^−4^	5.04 × 10^−3^	1.81 × 10^−4^
		3	6.98 × 10^−3^	2.16 × 10^−3^	2.16 × 10^−3^	1.51 × 10^−2^	5.43 × 10^−4^
		4	1.06 × 10^−2^	3.28 × 10^−3^	3.28 × 10^−3^	2.30 × 10^−2^	8.25 × 10^−4^
		5	1.81 × 10^−3^	5.60 × 10^−4^	5.60 × 10^−4^	3.92 × 10^−3^	1.41 × 10^−4^
		6	2.07 × 10^−3^	6.40 × 10^−4^	6.40 × 10^−4^	4.48 × 10^−3^	1.61 × 10^−4^
Cd	*Pheretima aspergillum* (E. Perrier)	1	9.41 × 10^−4^	5.88 × 10^−3^	2.35 × 10^−2^	5.67 × 10^−3^	1.57 × 10^−3^
		2	2.02 × 10^−4^	1.26 × 10^−3^	5.04 × 10^−3^	1.21 × 10^−3^	3.36 × 10^−4^
		3	4.48 × 10^−4^	2.80 × 10^−3^	1.12 × 10^−2^	2.70 × 10^−3^	7.47 × 10^−4^
		4	8.29 × 10^−4^	5.18 × 10^−3^	2.07 × 10^−2^	4.99 × 10^−3^	1.38 × 10^−3^
		5	4.03 × 10^−4^	2.52 × 10^−3^	1.01 × 10^−2^	2.43 × 10^−3^	6.72 × 10^−4^
		6	5.15 × 10^−4^	3.22 × 10^−3^	1.29 × 10^−2^	3.10 × 10^−3^	8.59 × 10^−4^
		7	2.46 × 10^−4^	1.54 × 10^−3^	6.16 × 10^−3^	1.48 × 10^−3^	4.11 × 10^−4^
	*Curcuma kwangsiensis* S. G. Lee et C. F. Liang	1	1.41 × 10^−4^	8.82 × 10^−4^	3.53 × 10^−3^	8.50 × 10^−4^	2.35 × 10^−4^
		2	5.24 × 10^−4^	3.28 × 10^−3^	1.31 × 10^−2^	3.16 × 10^−3^	8.74 × 10^−4^
		3	5.04 × 10^−4^	3.15 × 10^−3^	1.26 × 10^−2^	3.04 × 10^−3^	8.40 × 10^−4^
		4	9.68 × 10^−4^	6.05 × 10^−3^	2.42 × 10^−2^	5.83 × 10^−3^	1.61 × 10^−3^
		5	1.39 × 10^−3^	8.69 × 10^−3^	3.48 × 10^−2^	8.38 × 10^−3^	2.32 × 10^−3^
	*Oldenlandia diffusa* (Willd.) Roxb	1	1.14 × 10^−3^	7.14 × 10^−3^	2.86 × 10^−2^	6.88 × 10^−3^	1.90 × 10^−3^
		2	4.70 × 10^−4^	2.94 × 10^−3^	1.18 × 10^−2^	2.83 × 10^−3^	7.84 × 10^−4^
		3	6.72 × 10^−4^	4.20 × 10^−3^	1.68 × 10^−2^	4.05 × 10^−3^	1.12 × 10^−3^
		4	1.28 × 10^−3^	7.98 × 10^−3^	3.19 × 10^−2^	7.69 × 10^−3^	2.13 × 10^−3^
		5	4.03 × 10^−4^	2.52 × 10^−3^	1.01 × 10^−2^	2.43 × 10^−3^	6.72 × 10^−4^
		6	9.41 × 10^−4^	5.88 × 10^−3^	2.35 × 10^−2^	5.67 × 10^−3^	1.57 × 10^−3^
As	*Pheretima aspergillum* (E. Perrier)	1	2.58 × 10^−2^	1.29 × 10^−2^	2.58 × 10^−2^	8.59 × 10^−5^	/
		2	1.94 × 10^−2^	9.71 × 10^−3^	1.94 × 10^−2^	6.47 × 10^−5^	/
		3	8.44 × 10^−2^	4.22 × 10^−2^	8.44 × 10^−2^	2.81 × 10^−4^	/
		4	7.77 × 10^−2^	3.88 × 10^−2^	7.77 × 10^−2^	2.59 × 10^−4^	/
		5	4.74 × 10^−1^	2.37 × 10^−1^	4.74 × 10^−1^	1.58 × 10^−3^	/
		6	7.39 × 10^−2^	3.70 × 10^−2^	7.39 × 10^−2^	2.46 × 10^−4^	/
		7	6.72 × 10^−2^	3.36 × 10^−2^	6.72 × 10^−2^	2.24 × 10^−4^	/
	*Curcuma kwangsiensis* S. G. Lee et C. F. Liang	1	2.99 × 10^−2^	1.50 × 10^−2^	2.99 × 10^−2^	9.97 × 10^−5^	/
		2	6.38 × 10^−3^	3.19 × 10^−3^	6.38 × 10^−3^	2.13 × 10^−5^	/
		3	2.69 × 10^−3^	1.34 × 10^−3^	2.69 × 10^−3^	8.96 × 10^−6^	/
		4	3.29 × 10^−2^	1.65 × 10^−2^	3.29 × 10^−2^	1.10 × 10^−4^	/
		5	3.26 × 10^−2^	1.63 × 10^−2^	3.26 × 10^−2^	1.09 × 10^−4^	/
	*Oldenlandia diffusa* (Willd.) Roxb	1	1.12 × 10^−2^	5.60 × 10^−3^	1.12 × 10^−2^	3.73 × 10^−5^	/
		2	1.68 × 10^−2^	8.40 × 10^−3^	1.68 × 10^−2^	5.60 × 10^−5^	/
		3	1.23 × 10^−2^	6.16 × 10^−3^	1.23 × 10^−2^	4.11 × 10^−5^	/
		4	4.48 × 10^−3^	2.24 × 10^−3^	4.48 × 10^−3^	1.49 × 10^−5^	/
		5	3.36 × 10^−3^	1.68 × 10^−3^	3.36 × 10^−3^	1.12 × 10^−5^	/
		6	6.72 × 10^−3^	3.36 × 10^−3^	6.72 × 10^−3^	2.24 × 10^−5^	/

## Data Availability

All data generated or analyzed during this study are included in this published article.

## Abbreviations

TCM: traditional Chinese medicine; PBET: the physiologically based extraction test; ICP-MS: inductively coupled plasma mass spectrometry; FBS: fetal bovine serum; DMEM: Dulbecco’s modified eagle medium; TEER: transepithelial electrical resistance; HQ: hazard quotient; HI: hazard index; TTD: target organ toxicity dose
